# Association of DLT versus SLT with postoperative pneumonia during esophagectomy in China: a retrospective comparison study

**DOI:** 10.1186/s12871-023-02252-4

**Published:** 2023-09-05

**Authors:** Xukeng Guo, Weiqi Ke, Xin Yang, Xinying Zhao, Meizhen Li

**Affiliations:** Department of Anesthesiology, the First Affiliated Hospital of Shantou University Medical College, No. 57 Changping Road, Jinping District, Shantou City, Guangdong Province China

**Keywords:** Esophageal cancer, Double lumen tube, Single lumen tube, Postoperative, Pneumonia

## Abstract

**Background:**

Double lumen tube (DLT) and single lumen tube (SLT) are two common endotracheal tube (ETT) types in esophageal cancer surgery. Evidence of the relationship between two ETT types and postoperative pneumonia (PP) remains unclear. We aimed to determine the association between two types of ETT (DLT and SLT) and PP and assess the perioperative risk-related parameters that affect PP.

**Methods:**

This study included 680 patients who underwent esophageal cancer surgery from January 01, 2010 through December 31, 2020. The primary outcome was PP, and the secondary outcome was perioperative risk-related parameters that affect PP. The independent variable was the type of ETT: DLT or SLT. The dependent variable was PP. To determine the relationship between variables and PP, univariate and multivariate analyses were performed. The covariables included baseline demographic characteristics, comorbidity disease, neoadjuvant chemotherapy, tumor location, laboratory parameters, intraoperative related variables.

**Results:**

In all patients, the incidence of postoperative pneumonia in esophagectomy was 32.77% (36.90% in DLT group and 26.38% in SLT group). After adjusting for potential risk factors, we found that using an SLT in esophagectomy was associated with lower risk of postoperative pneumonia compared to using a DLT (Odd ratio = 0.41, 95% confidence interval (CI): 0.22, 0.77, *p* = 0.0057). Besides DLT, smoking history, combined intravenous and inhalation anesthesia (CIIA) and vasoactive drug use were all significant and independent risk factors for postoperative pneumonia in esophagectomy. These results remained stable and reliable after subgroup analysis.

**Conclusions:**

During esophagectomy, there is significant association between the type of ETT (DLT or SLT) and PP. Patients who were intubated with a single lumen tube may have a lower rate of postoperative pneumonia than those who were intubated with a double lumen tube. This finding requires verification in follow-up studies.

**Supplementary Information:**

The online version contains supplementary material available at 10.1186/s12871-023-02252-4.

## Introduction

Esophageal cancer remains one of the major causes of cancer mortality and burden worldwide [1]. Esophagectomy is a critical treatment for esophageal cancer [2]. Postoperative pneumonia (PP) is the most common complication after esophageal cancer surgery, with an incidence ranging from 17.7% to 38% [3–7]. In fact, PP raises hospital costs, lengthens hospital stays, and raises the risk of death [8]. As a result, lowering the incidence of PP is extremely important clinically.

As reported, among the high risk factors for PP are age, tumor site, nutrition, pulmonary function, types of operation, types of endotracheal tube (ETT) anesthesia and the modes of ventilation etc [2, 9, 10]. It is revealed that the types of ETT anesthesia and the modes of ventilation correlated with the incidence of PP in esophageal cancer surgery [11–14]. According to the types of endotracheal tube and operation request, ETT in esophagectomy is divided into the double lumen tube (DLT) and the single lumen tube (SLT). Some studies found that the incidence of PP between these two types of ETT was similar [11, 13, 15]. However, another study from R. Souche et al. demonstrated that using a SLT to achieve two lung ventilation (TLV) mode could reduce the incidence of PP compare to using a DLT [14]. It seems that the effects of ETT types on incidence of PP still remains controversial.

Based on previous research on ETT type and PP, we aim to determine whether SLET is associated with lower rate of PP in esophagectomy.

## Materials and methods

### Study design and participants

With approval from the Ethics Committee of the First Affiliated Hospital of Shantou University Medical College (NO. B-2021–249), this retrospective cohort study collected all the medical records of patients who underwent radical esophageal malignant tumor resection of the First Affiliated Hospital of Shantou University Medical College in Guangdong, China, between January 01, 2010 and December 31, 2020. Patients with an unplanned second surgery, cancelled operation, or combined surgery with other sites, against-advice discharge or postoperative death, and non-esophageal cancer after postoperative pathological examination or missing medical records were excluded. The data was analyzed anonymously, and the requirement for informed consent was waived. This study complied with the Declaration of Helsinki and adhered to the applicable STROBE guidelines.

### Surgical and anesthetic techniques options

Esophagectomy surgical techniques are divided into two types: open esophagectomy (OE) and minimally invasive esophagectomy (MIE). Based on the anastomotic site, MIE procedures were divided into two types: Mckeown MIE with anastomosis in the neck (thoracoscopic esophagectomy and laparoscopic gastric mobilisation with cervical anastomosis) and Lvor-Lewis MIE with anastomosis in the chest (a thoracic phase with esophagectomy and intrathoracic esophagogastric anastomosis). Generally, esophageal tumors in the upper and middle thoracic segments were appropriate for McKeown MIE, whereas those in the lower thoracic segment were better suited for Ivor-Lewis MIE or OE. The most prevalent surgical methods in our hospital are the OE (left or right transthoracic surgery) and the Mckeown MIE (the right transthoracic procedure). Lvor-Lewis MIE is rarely performed since it is difficult not only to remove the lymph nodes surrounding the left recurrent laryngeal nerves, but also to control the progression of anastomotic leaking after it has occurred. In summary, Meckeown MIE is favored in the majority of instances. When the tumor is close to the stomach cardia, surgeons will choose the OE surgery. For esophagectomy, anesthesiologists may use general anesthesia (GA) or a combination of GA and thoracic epidural (E-GA). After routine general anesthesia induction, a DLT was placed in the OE technique to decompress the left lung, or a single lumen endotracheal intubation was performed in the MIE operation for two lung ventilation [16]. However, if the patient who planning to place a DLT and presents a difficult airway, bronchial blockers could be considered for one lung ventilation after awake intubation with fiber optic bronchoscopy through a SLT [17]. The anesthesia plan is developed by the anesthesiologist in consultation with the surgeon and the patient after a thorough preoperative evaluation of the patient. Perioperative management is individualized for each patient by the anesthesiologist.

### Outcomes and variables

The primary outcome was the incidence of PP between DLT and SLT group and the secondary outcome was perioperative risk-related parameters that affect PP. The diagnoses of postoperative pneumonia depend on clinical symptoms and imaging within the first two weeks after esophagectomy: (1) with clinical symptoms of cough, productive cough, fever or chest tightness, leukocyte count > 10.0 × 10^9^ /L or < 4.0 × 10^9^ /L, and purulent secretions; (2) postoperative imaging of new or progressive development, persistent pulmonary infiltrate shadows, consolidation, or cavitation [18].

In addition to the target independent variables (SLT and DLT) and the dependent variable (postoperative pneumonia), we included the following covariables, which are perioperative risk-related factors that affect postoperative pneumonia, as described below: 1) Baseline demographic characteristics (age, gender, smoking and drinking status); 2) Comorbidity disease (hypertension, diabetes, or pulmonary disease); 3) Neoadjuvant chemotherapy and tumor location; 4) Laboratory inspection results (hemoglobin (Hb), albumin (ALB)); 5) Intraoperative related variables (American Society of Anesthesiologists Physical Status (ASA), type of anesthesia (general anesthesia (GA), combined epidural-general anesthesia (E-GA)), continuous anesthesia(total intravenous anesthesia (TIVA), combined intravenous and inhalation anesthesia (CIIA)), surgery method (open esophagectomy (OE), minimally invasive esophagectomy (MIE)), vasoactive drug use, operation time (OT), perioperative fluid volume (PFV), estimated blood loss (EBL)), patient controlled analgesia (PCA) (patient controlled intravenous analgesia (PCIA), patient controlled epidural analgesia (PCEA)).

### Study size and power calculation

The power of sample size was estimated by PASS 15.0 (NCSS, Kaysville, UT, USA). The incidences of postoperative pneumonia between DLT and SLT group were 36.90% vs.26.38%, respectively. Assuming an alpha error of 0.05 (two-sided) and at the end, the sample sizes between DLET and SLET were 393 vs.254, respectively. In our study, the power was calculated to be approximately 81%. Thus, this study was sufficiently powerful when compared to a power of 0.8 that was estimated when we designed the study conventionally.

### Statistical analyses

Descriptive statistics were used to characterize patient demographic and clinical data. Continuous data were expressed as mean standard deviation (normal distribution) or medians with interquartile ranges (nonnormal distribution), and categorical variables were presented as percentages. For determining the normality of continuous variables, the Kolmogorov–Smirnov test was applied. The two sample t test was used to evaluate continuous data with a normal distribution, whereas the Mann–Whitney U test was used to investigate continuous variables with a nonnormal distribution. To evaluate categorical variables, the Chi-squared or Fisher's exact test was used.

Univariate logistic regression was used to find out the relationship between candidate variables that are perioperative risk factors and postoperative pneumonia after esophageal cancer surgery. Variables were selected as candidates for multivariable analysis based on the level of significance of the bivariate association (*P* < 0.05). Using multivariate logistic regression, the association between the candidate variables and postoperative pneumonia was investigated, and the odds ratio (OR) and 95% confidence interval (CI) for the risk of postoperative pneumonia between patients with DLET and those with SLET were calculated. The variance inflation factor (VIF) was used to assess multicollinearity between ETT and all other independent variables, with VIF greater than 10 considered suggestive of multicollinearity [19]. These covariables were included in the final model if they changed the estimate of the dependent variable ETT type on postoperative pneumonia by more than 10% or if they were significantly related to postoperative pneumonia. The details of collinearity analysis and the associations of each covariables with outcomes of postoperative pneumonia were shown in Supplementary Tables 1–4.

Three multivariate logistic regression models were constructed: 1) Crude (unadjusted); 2)Model I (minimally adjusted): adjusted for baseline demographic variables that were the risk factors of postoperative pneumonia: age, gender, smoking history, drinking history, pulmonary diseases; 3) Model II(fully adjusted): adjusted for variables related risk factors of postoperative pneumonia and changed the estimate of dependent variable ETT type on postoperative pneumonia more than 10%: age, gender, smoking history, drinking history, pulmonary diseases, neoadjuvant chemotherapy, type of anesthesia, continuous anesthesia, vasoactive drug use, surgery method, OT, EBL.

Subgroup analysis was employed using a stratified multivariate logistic regression across various subgroups. First, we transformed the continuous variable into categorical variables: age (< 60, ≥ 60 years), Hb (< 130/125, ≥ 130/125 g/L), ALB (< 35, ≥ 35 g/L), OT (≤ 240, > 240 min), PFV (≤ 2000, > 2000 ml) and EBL (≤ 200, > 200 ml). Second, besides the stratification factor itself, we also adjusted each stratification for all factors (age, sex, smoking, drinking, hypertension, diabetes, pulmonary diseases, neoadjuvant chemotherapy, anemia (male: Hb < 130 g/L, female: Hb < 125 g/L), ALB, ASA Status, tumor location, type of anesthesia, type of ETT, continuous anesthesia, vasoactive drug use, surgery method, OT, PFV, EBL, PCA). Finally, tests for interaction were applied to the likelihood ratio test of models with and without interaction terms.

Data were analyzed using the R package, version 3.4.3 (http://www.r-project.org) and Empower Stats (https://www.empowerstats.net/cn/; X&Y Solutions, Inc., Boston, MA, USA). Prism 9.0 (GraphPad Software, La Jolla, CA, USA) were utilized to draw the figure. P values for two-sided < 0.05 were considered statistically significant.

## Results

### Study participants

**Fig. 1:**
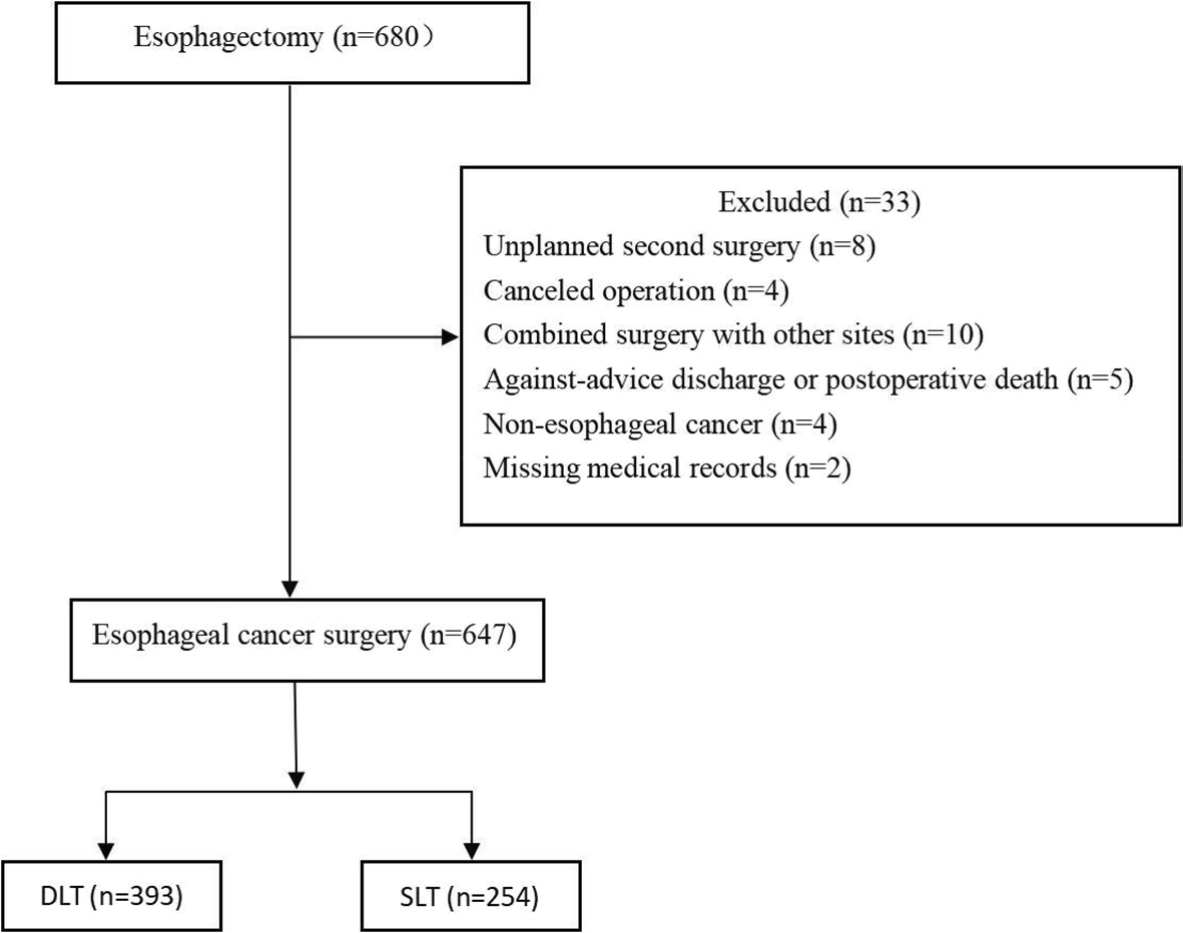
Study population flow diagram

A total of 680 patients were identified in this study who underwent esophagectomy between January 1, 2010 and December 31, 2020, with 33 being excluded due to: unplanned second surgery (*n* = 8); cancellation of operation (*n* = 4); multi-site combined surgery (*n* = 10); unplanned discharge or postoperative death (*n* = 5); postoperative pathological diagnosis of non-tumor (*n* = 4); and medical records loss (*n* = 2). Therefore, 647 patients (393 with DLT and 254 with SLT) were included in this study's final analysis (Fig. 1).

### Baseline demographic and clinical characteristics of participants

Table 1 summarizes the demographic and clinical characteristics of groups DLT and SLT. The covariables were unequally distributed between the two groups. The SLT group (62.61 ± 7.71 years old) was older than the DLT group (60.06 ± 8.30 years old) (*P* < 0.001). As shown, the following confounders were more prevalent in the DLT group than in the SLT group: smoking history, E-GA, TIVA, PECA, OE, and EBL (> 200 ml) (*P* < 0.05). Participants in the SLT group had a higher proportion of hypertension, neoadjuvant chemotherapy, vasoactive drug use, and OT. Postoperative pneumonia in esophagectomy was 36.90% in the DLT group and 26.38% in the SLT group.

**Table 1:** Baseline demographic and clinical characteristics of participants

Type of endotracheal tube	Total	DLT	SLT	*P* Value
N	647	393	254	
Preoperative				
Age, mean (SD), y	61.06 (8.16)	60.06 (8.30)	62.61(7.71)	< 0.001
Gender, n (%)				0.062
Female	146 (22.57)	79(20.10)	67(26.38)	
Male	501 (77.43)	314(79.90)	187 (73.62)	
Smoking, n (%)	362 (55.95)	244 (57.00)	118 (46.46)	0.009
Drinking, n (%)	189 (29.21)	121 (30.79)	68(26.77)	0.273
Hypertension, n (%)	102 (15.77)	51(12.98)	51 (20.08)	0.015
Diabetes, n (%)	47 (7.26)	25 (6.36)	22 (8.66)	0.271
Pulmonary diseases, n (%)	150 (23.18)	100 (25.45)	50 (19.69)	0.09
Neoadjuvant chemotherapy, n (%)	142 (21.94)	59 (15.01)	83 (32.68)	< 0.001
Hb, mean (SD), g/L	130.87 (15.10)	131.60 (14.04)	129.73(16.59)	0.194
ALB, mean (SD), g/L	39.63 (4.24)	39.97 (4.05)	39.12 (4.47)	0.273
ASA Status, n (%)				0.341
1	20 (3.09)	14 (3.56)	6 (2.36)	
2	574 (88.72)	351 (89.31)	223 (87.80)	
3	53 (8.19)	28 (7.12)	25 (9.84)	
Tumor location, n (%)				0.845
Upper	44(6.80)	28 (7.12)	16 (6.30)	
Middle	484(74.81)	291 (74.05)	193 (75.98)	
Lower	119(18.39)	74 (18.83)	45 (17.72)	
Intraoperative				
Type of anesthesia, n (%)				< 0.001
E-GA	462(71.41)	341(86.77)	121 (47.64)	
GA	185(28.59)	52(13.23)	133 (52.36)	
Continuous anesthesia, n (%)				0.004
TIVA	587(90.73)	367(93.38)	220 (86.61)	
CIIA	60(9.27)	26 (6.62)	34 (13.39)	
Vasoactive drug use, n (%)	347(52.70)	165 (41.98)	176 (69.29%)	< 0.001
Surgery method, n (%)				< 0.001
OE	347(53.63)	338 (86.01)	9 (3.54)	
MIE	300(46.37)	55 (13.99)	245 (96.46)	
OT, mean (SD), mins,	239.14 (56.67)	225.48 (58.41)	260.26 (46.79)	< 0.001
PFV, n (%), ml				0.432
≤ 2000	146(22.57)	90(30.72)	56(27.45)	
> 2000	351(54.25)	203(69.28)	148(72.55)	
EBL, n (%), ml				< 0.001
≤ 200	413(63.83)	183(46.56)	230(90.55)	
> 200	234(36.16)	210(53.44)	24(9.45)	
PCA, n (%)				< 0.001
PCIA	188(29.06)	52 (13.23)	136 (53.54)	
PCEA	459(70.94)	341 (86.77)	118 (46.46)	
Postoperative pneumonia, n (%)	212(32.77)	145 (36.90)	67 (26.38)	0.005

*Abbreviation*: *DLT* Double lumen tube, *SLT* Single lumen tube, *Hb* Hemoglobin, *ALB* Albumin, *ASA* American Society of Anesthesiologist, *E-GA* Combined epidural-general anesthesia, *GA* General anesthesia, *TIVA* Total intravenous anesthesia, *CIIA* Combined intravenous and inhalation anesthesia, *OE* Open esophagectomy, *MIE* Minimally invasive esophagectomy, *OT* Operation time, *PFV* Perioperative fluid volume, *EBL* Estimated blood loss, *PCA* Patient controlled analgesia, *PCIA* Patient controlled intravenous analgesia, *PCEA* Patient controlled epidural analgesia

### Univariate and multivariate analysis

**Fig. 2:**
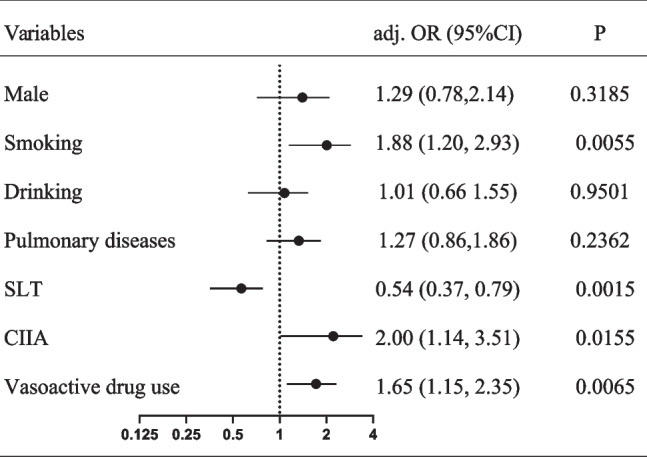
Multivariable logistic regression analysis for the independent effects of male, smoking history, drinking history, pulmonary diseases, TIVA, vasoactive drug use on the risk of postoperative pneumonia. As all VIF values were < 10 (maximal VIF was 4.8), we confirmed the absence of multicollinearity. Abbreviation: adj, adjusted; SLT, single lumen tube; TIVA, total intravenous anesthesia; OR, odds ratio; CI, confidence interval; VIF, variance inflation factor

Male, smoking history, drinking history, pulmonary diseases, DLT, CIIA, and vasoactive drug use were statistically significant risk factors for postoperative pneumonia in the univariate logistic regression analysis (Table 2). After adjusting for other covariates, smoking history, DLT, CIIA, and vasoactive drug use were identified as independent risk factors for postoperative pneumonia in a multivariable analysis (Fig. 2).

**Table 2:** Univariate analysis for postoperative pneumonia of patients

	Statistics	OR (95%CI)	*P* value
Age, mean (SD), y	61.06 (8.16)	1.02 (1.00, 1.04)	0.082
Age, n (%), y			
> 60	274 (42.35)	Reference	
≥ 60	373 (57.65)	1.22 (0.87, 1.70)	0.2507
Gender, n (%)			
Female	146 (22.57)	Reference	
Male	501 (77.43)	2.00 (1.30, 3.08)	0.0017
Smoking, n (%)	342 (52.86)	2.31 (1.64, 3.25)	< 0.0001
Drinking, n (%)	189 (29.21)	1.64 (1.16, 2.34)	0.0057^*^
Hypertension, n (%)	102 (15.77)	1.03 (0.66, 1.62)	0.8943
Diabetes, n (%)	47 (7.26)	0.61 (0.30, 1.22)	0.1593
Pulmonary diseases, n (%)	150 (23.18)	1.57 (1.08, 2.29)	0.0192
Neoadjuvant chemotherapy, n (%)	142 (21.95)	1.30 (0.88, 1.91)	0.191
Hb, n (%), g/L	130.87 (15.11)	1.00 (0.99, 1.01)	0.9491
Anemia, n (%)	237 (36.63)	1.15 (1.01, 1.23)	0.3531
ALB, mean (SD), g/L	39.63 (4.24)	0.98 (0.94, 1.02)	0.2932
ALB, n (%), g/L			
< 35	69 (10.66)	Reference	
≥ 35	578 (89.34)	0.73 (0.44, 1.22)	0.2348
ASA Status, n (%)			
1	20 (3.09)	Reference	
2	574 (88.72)	0.56 (0.23, 1.37)	0.2036
3	53 (8.19)	0.94 (0.33, 2.64)	0.902
Tumor location, n (%)			
Upper	44 (6.80)	Reference	
Middle	484 (74.81)	0.61 (0.33, 1.14)	0.1198
Lower	119 (18.39)	0.67 (0.33, 1.35)	0.2605
Type of anesthesia, n (%)			
E-GA	462 (71.41)	Reference	
GA	185 (28.59)	0.91 (0.63, 1.32)	0.6275
Type of ETT, n (%)			
DLT	393 (60.74)	Reference	
SLT	254 (39.26)	0.61 (0.43, 0.87)	0.0056
Continuous anesthesia, n (%)			
CIIA	60 (9.27)	Reference	
TIVA	587 (90.73)	0.52 (0.31, 0.89)	0.0175
Vasoactive drug use, n (%)	341 (52.70)	1.46 (1.05, 2.03)	0.0263
Surgery method, n (%)			
MIE	300 (46.37)	Reference	
OE	347 (53.63%)	1.30 (0.93, 1.81)	0.1186
OT, n (%), mins	239.13 (56.72)	1.00 (1.00, 1.00)	0.6086
OT, n (%), mins			
≤ 240	366 (56.57)	Reference	
> 240	281 (43.43)	1.03 (0.74, 1.43)	0.8757
PFV, n (%), ml			
≤ 2000	202 (31.22)	Reference	
> 2000	445 (68.78)	1.04 (0.73, 1.48)	0.8299
EBL, n (%), ml			
≤ 200	413 (63.83)	Reference	
> 200	234 (36.17)	1.07 (0.76, 1.51)	0.6851
PCA, n (%)			
PCEA	459 (70.94)	Reference	
PCIA	188 (29.06)	0.85 (0.59, 1.23)	0.3962

*Abbreviation*: *DLT* Double lumen tube, *SLT* Single lumen tube, *Hb* Hemoglobin, *ALB* Albumin, *ASA* American Society of Anesthesiologist, *E-GA* Combined epidural-general anesthesia, *GA* General anesthesia, *TIVA* Total intravenous anesthesia, *CIIA* Combined intravenous and inhalation anesthesia, *OE* Open esophagectomy, *MIE* Minimally invasive esophagectomy, *OT* Operation time, *PFV* Perioperative fluid volume, *EBL* Estimated blood loss, *PCA* Patient controlled analgesia, *PCIA* Patient controlled intravenous analgesia, *PCEA* Patient controlled epidural analgesia

We performed three models to evaluate the independent correlation between two types of ETT and incidence of postoperative pneumonia. Table 3 displays the effect sizes (odd ratio, OR) and 95% confidence intervals (95% CI). The incidence of postoperative pneumonia was 38% lower with SLT compared with DLT in the crude model (unadjusted model) (OR = 0.62, 95% CI:0.44–0.87, *P* = 0.0063). The risk ratios for postoperative pneumonia in individuals with SLT were 0.55 (95% CI: 0.38, 0.81) in model I (minimally-adjusted model). Furthermore, in model II (fully adjusted model), the odds ratio for postoperative pneumonia in individuals with SLT was 0.41 (95% CI: 0.22, 0.77) compared to DLT.

**Table 3:** Relationship between two types of endotracheal tube and postoperative pneumonia

Outcome	Crude Model		Model I		Model II	
	OR (95%CI)	*P*-value	OR (95%CI)	*P*-value	OR (95%CI)	*P*-value
DLT	Reference		Reference		Reference	
SLT	0.62 (0.44, 0.87)	0.0063	0.55 (0.38, 0.81)	0.0022	0.41(0.22, 0.77)	0.0057

Crude (unadjusted) adjust for: None

Model I (minimally adjusted) adjust for: age, gender, smoking, drinking, pulmonary diseases

Model II (fully adjusted) adjust for: age; gender; smoking; drinking; hypertension; pulmonary diseases; neoadjuvant chemotherapy; type of anesthesia; continuous anesthesia; vasoactive drug use; surgery method; OT; EBL

*Abbreviation*: *OR* Odds ratio, *CI* Confidence interval, *DLT* Double lumen tube, *SLT* Single lumen tube, *OT* Operation time, *EBL* Estimated blood loss

### Subgroup analysis

All categorical variables (gender, smoking, drinking, hypertension, diabetes, pulmonary diseases, neoadjuvant chemotherapy, anemia, ASA Status, tumor location, type of anesthesia, type of ETT, continuous anesthesia, vasoactive drug use, surgery method, PCA) and continuous variables (age, ALB, OT, PFV, EBL) were transformed into categorical variables. The subgroup analysis shown in Table 4 revealed that there were no substantially different interactions in any of the variables (all *p-value* > 0.05).

**Table 4:** Subgroup analysis of patients

Variables	DLT	SLT	RR (95%CI)	*P* value for interaction
	Pneumonia (%)	Pneumonia (%)		
Total	145/393 (36.90)	67/254 (26.38)	0.71 (0.51, 1.01)	-
Age				0.4666
< 60	66/191 (34.55)	17/83 (20.48)	0.59 (0.32, 1.09)	
≥ 60	79/202 (39.11)	50/171 (29.24)	0.75 (0.48, 1.15)	
Gender				0.9625
Female	20/79 (25.32)	12/67 (17.91)	0.71 (0.32, 1.58)	
Male	125/314 (39.81)	55/187 (29.41)	0.74 (0.50, 1.09)	
Smoking				0.291
Yes	103/224 (45.98)	38/118 (32.20)	0.70 (0.44, 1.12)	
No	42/169 (24.85)	29136 (21.32)	0.86 (0.50, 1.47)	
Drinking				0.9404
Yes	59/121 (48.76)	23/68 (33.82)	0.69 (0.37, 1.28)	
No	91/272 (33,46)	44/186 (23.66)	0.71 (0.46, 1.08)	
Hypertension				0.0774
No	129/342 ((37.72)	49/203 (24.14)	0.64 (0.43, 0.94)	
Yes	16/51 (31.37)	18/51 (35.29)	1.13 (0.49, 2.57)	
Diabetes				0.5419
No	139/358 (37.77)	62/232 (26.72)	0.71 (0.49, 1.01)	
Yes	6/25 (24.00)	5/22 (22.73)	0.95 (0.24, 3.67)	
Pulmonary diseases				0.884
No	101/293 (34.47)	50 (24,51)	0.71 (0.48, 1.06)	
Yes	44/100 (44.00)	17/50 (34.00)	0.77 (0.38, 1.57)	
Neoadjuvant chemotherapy				0.0703
Yes	31/59 (52.54)	22/83 (23.51)	0.50 (0.25, 1.02)	
No	114/334 (34.13)	45/171 (26.32)	0.77 (0.51, 1.16)	
Anemia				0.0153
Yes	62/140 (44.29)	21/97(21.65)	0.49 (0.27, 0.88)	
No	83/252 (32.94)	46/157 (29.30)	0.89 (0.58, 1.37)	
ALB(g/L)				0.2612
< 35	20/41 (48.78)	7/28 (25.00)	0.51 (0.18, 1.47)	
≥ 35	125/352 (35.51)	60/226 (26.55)	0.75 (0.52, 1.08)	
ASA Status				0.1734
1	6/14 (42.86)	3/6 (50.00)	1.17 (0.17, 7.95)	
2	128/351 (36.48)	52/223 (23.32)	0.64 (0.44, 0.93)	
3	11/28 (39.26)	12/24 (50.00)	1.27 (0.42, 3.83)	
Tumor location				0.8596
Upper	13/28 (46.43)	6/16 (37.50)	0.81 (0.23, 2.83)	
Middle	103/291 (35.40)	50/193 (25.91)	0.73 (0.49, 1.09)	
Lower	29/74 (39.19)	11/45 (24.44)	0.62 (0.27, 1.42)	
Type of anesthesia				0.3863
E-GA	23/52 (43.40)	35/133 (26.32)	0.59 (0.30, 1.16)	
GA	122/341 (35.78)	32/121 (26.45)	0.74 (0.47, 1.17)	
Continuous anesthesia				0.1074
CIIA	17/26 (65.38)	11/34 (32.35)	0.49 (0.17, 1.46)	
TIVA	128/367 (34.88)	56/220 (25.45%)	0.73 (0.50, 1.06)	
Vasoactive drug use				0.9509
Yes	73/165 (44.24)	52/176 (29.55)	0.67 (0.43, 1.04)	
No	72/228 (31.58)	15/78 (19.23)	0.61 (0.32, 1.14)	
Surgery method				0.409
MIE	23/55 (41.82)	66/245 (26.94)	0.64 (0.35, 1.18)	
OE	122/338 (36.09)	1/9 (11.11)	0.31 (0.04, 2.49)	
OT (min)				0.5114
≤ 240	95/273 (34.80)	24/93 (25.81)	0.74 (0.44, 1.26)	
> 240	50/120 (41.67)	43/161 (26.71)	0.64 (0.39, 1.06)	
PFV (ml)				0.5237
≤ 2000	48/129 (37.21)	17/73 (23.29)	0.63 (0.33, 1.20)	
> 2000	97/264 (36.74)	50/181 (27.62)	0.75 (0.50, 1.13)	
EBL (ml)				0.8284
≤ 200	72/183 (39.34)	61/230 (26.52)	0.67 (0.44, 1.02)	
> 200	73/216 (33.80)	6/24 (25.00)	0.74 (0.28, 1.94)	
PCA				0.1028
PCEA	121/341 (35.48)	34/118 (28.81)	0.81 (0.51, 1.28)	
PCIA	24/52 (46.15)	33/136 (24.26)	0.53 (0.27, 1.03)	

*Abbreviation*: *RR* Rate ratio, *CI* Confidence interval, *DLT* Double lumen tube, *SLT* Single lumen tube, *Hb* Hemoglobin, *ALB* Albumin, *ASA* American Society of Anesthesiologist, *E-GA* Combined epidural-general anesthesia, *GA* General anesthesia, *TIVA* Total intravenous anesthesia, *CIIA* Combined intravenous and inhalation anesthesia, *OE* Open esophagectomy, *MIE* Minimally invasive esophagectomy, *OT* Operation time, *PFV* Perioperative fluid volume, *EBL* Estimated blood loss, *PCA* Patient controlled analgesia, *PCIA* Patient controlled intravenous analgesia, *PCEA* Patient controlled epidural analgesia

## Discussion

In this study, we retrospectively discovered high-risk factors for PP and assessed the relationship between two types of ETT and PP in patients underwent esophageal cancer surgery from January 01,2010 to December 31, 2020. Smoking history, DLT, CIIA, and vasoactive medication usage were all significant and independent risk factors for postoperative pneumonia in patients undergoing esophageal cancer surgery, according to our findings. Furthermore, three models were constructed to clarify the effect of ETT type on PP after adjusting for potential risk factors. The findings revealed that the risk of PP was considerably lower in the SLT group compared to the DLT group. Moreover, subgroup and interaction analyses demonstrated that the relationship between SLT and a lower risk of PP was not modified by any covariables.

PP in esophagectomy was 36.90% in the DLT group and 26.38% in the SLT group. Both incidence rates of PP are relatively high when compared to other studies, but these findings are based on real-world data and cannot be denied. According to reports, the incidence of PP after esophageal cancer surgery ranges from 17.7% to 38% [3–7]. The incidence rates of PP are high but still within a reasonable range. Our study demonstrated the SLT anesthesia in esophagectomy could significantly reduce the risk of PP compared with the DLT anesthesia. This finding was consistent with a multicenter case–control study of 137 patients [14]. However, Miao Lin et al. found no differences in the incidence of PP between SLET and DLT in a study of 1166 patients [11], which was similar to what Lei Cai and his colleagues discovered [13]. The possible explanation for this result might be that Miao Lin et al. did not analyze the relationship between two types of ETT and PP independently or adjust the covariables, although its sample size was bigger. According to the study by Lei Cai et al., the incidence of PP was 4.8% in patients with SLT and 7.4% in patients with DLT. It was not statistically significant, but it was significant in clinical situations. In other words, when compared to DLT, using a SLT reduced the risk of PP by 36%. It is possible that the sample size was insufficient to detect a difference in PP between two types of ETT.

The reasons why using a SLT could reduce the risk of PP compared to the DLT are as follows. First, SLT achieves TLV, promotes oxygenation in lung and reduces intrapulmonary shunt. Second, DLT for OLV may result in ischemia–reperfusion and hypoxia-reoxygenation injuries, as well as bilateral inflammatory response [20]. As a result, patients with DLT have a higher risk of PP than those with SLT. In clinical practice, under the premise of ensuring adequate ventilation, adequate oxygenation and to guarantee the patency of the airway, the anesthesiologists choose the appropriate endotracheal tube type as possible to meet the requirement of surgeons, which helps reduce the incidence of postoperative pulmonary complications.

Our findings show that, in addition to DLT, smoking history, CIIA, and vasoactive drug use are significant and independent risk factors for PP in patients undergoing esophageal cancer surgery. Smoking for an extended period of time damages the ciliary structure of the airway mucosa, reducing its ability to clear mucus. Smoking patients are more likely than nonsmokers to develop airway obstruction and pulmonary infection [21–24]. According to a systematic review and meta-analysis, quitting smoking for 4–8 weeks before surgery can reduce the risk of postoperative pulmonary complications by 23%-47% [25]. Patients with CIIA had a higher risk of PP than those with TIVA in this study. However, a recent clinical trial found no significant difference in postoperative pulmonary complications when using volatile anesthetics sevoflurane or desflurane versus intravenous anesthetic propofol in lung surgery [26]. It is still debatable whether volatile anesthetics can reduce PP when compared to intravenous anesthetics [27, 28]. Vasoactive drugs are commonly used perioperatively in esophageal cancer surgery. As previously reported, the use of vasoactive drugs could reduce postoperative complications and length of hospital stay in abdominal surgery [29], which was opposite to what we found in esophagectomy. A possible reason might be that the effect of fluid infusion volume on PP during the esophageal cancer surgery is uncertain [30]. In this study, the vasoactive drug was used to maintain blood pressure, but the intraoperative fluid administration was similar between two groups. Patients with vasoactive drugs may have fluid volume deficits, which will influence the pulmonary circulation, causing PP. Therefore, smoking cessation before surgery, using correct anesthetic drugs during surgery, and mastering the correct application time of vasoactive drug use may be beneficial to reduce the occurrence of PP. Anesthesiologist is the central role during peri-operative. We should have a different approach to a different patient "tailored" peri-operative approach to esophagectomy [16].

This is the first study to independently analyze the relationship between the two types of ETT and PP in esophageal cancer surgery. Although this retrospective cohort study did not have the largest sample size when compared to other studies, it did have sufficient power of sample size. Strictly statistical adjustments were employed to minimize residual confounders. Furthermore, we constructed three different models and the results consistently revealed that SLT anesthesia in esophagectomy reduced the incidence of PP than DLT anesthesia, which was stable and reliable. However, there are still some limitations in this paper. First, it is a single-center study, therefore the results may not be applicable to other centers. Second, the time point of onset of PP was not included in the analyze because of the missing data. According to D’journo et al., the major respiratory complications after open esophagectomy occurred in 30% of patients, with the majority occurring during the first five days of surgery [31]. Third, the study period in this retrospective cohort study is relatively long. The use of endotracheal tube types varied from year to year. However, the annual incidence of pneumonia in group DLT was still higher than that in group SLT, it did not affect our findings (Supplementary Tables 5, 6, Supplementary Fig. 1). Fourth, there was a significant difference in the esophagectomy approach between the groups: most patients underwent MIE in the SLT group, whereas 86% did OE in the DLT group. However, the incidence of PP was significantly fewer in the SLT group irrespective of the operative approach (Table 4). Fifth, the improvement of perioperative management is a potential confounder that cannot be quantified and there are other un unknown confounders, except for the included confounders.

## Conclusions

During esophagectomy, there is significant association between the type of ETT (DLT or SLT) and PP. Patients with SLT anesthesia may have a lower incidence of PP than those with DLT anesthesia in esophageal cancer surgery. Anesthesiologists should choose the most appropriate type of ETT for anesthesia after comprehensive evaluation for the surgery. This result needs to be further validated by other multi-center research in the future.

### Supplementary Information

**Additional file 1: Supplementary Table 1.** Independent variables collinearity check (VIF selection). **Supplementary Table 2.** Univariate analysis of covariates vs postoperative pneumonia. **Supplementary Table 3.** Add covariates to basic model or remove it from full model, check coeff of X X= Type of endotracheal tube. **Supplementary Table 4.** Selected covariates. 

**Additional file 2: Supplementary Table 5.** A timetable on the use of different endotracheal tube. **Supplementary Table 6.** The detail of timetable on the use of different endotracheal tube and the occurrence of pneumonia. **Supplementary Figure 1.** Pneumonia in two groups from 2010 to 2020.

## Data Availability

The datasets generated and analyzed during the current study are not publicly available due to institutional restrictions but are available from the corresponding author on reasonable request.

## Abbreviations

PP: Postoperative pneumonia
ETT: Endotracheal tube
DLT: Double lumen tube
SLT: Single lumen tube
OE: Open esophagectomy
MIE: Minimally invasive esophagectomy
GA: General anesthesia
E-GA: Combined epidural-general anesthesia
Hb: Hemoglobin
ALB: Albumin
ASA: American Society of Anesthesiologist Physical Status
TIVA: Total intravenous anesthesia
CIIA: Combined intravenous and inhalation anesthesia
OT: Operation time
PFV: Perioperative fluid volume
EBL: Estimated blood loss
PCA: Patient controlled analgesia
PCIA: Patient controlled intravenous analgesia
PCEA: Patient controlled epidural analgesia
CI: Confidence intervals
