# Supplementary material for: Association of DLT versus SLT with postoperative pneumonia during esophagectomy in China: a retrospective comparison study
Source: BMC Anesthesiol. 2023 Sep 5;23:301. doi: 10.1186/s12871-023-02252-4 (PMC10478392; doi:10.1186/s12871-023-02252-4)
Supplement: Supplementary file 1 — Additional file 1: Supplementary Table 1. Independent variables collinearity check (VIF selection). Supplementary Table 2. Univariate analysis of covariates vs postoperative pneumonia. Supplementary Table 3. Add covariates to basic model or remove it from full model, check coeff of X X= Type of endotracheal tube. Supplementary Table 4. Selected covariates. [file 12871_2023_2252_MOESM1_ESM.docx]

Supplementary table 1. Independent variables collinearity check (VIF selection):

| Variables | VIF |
| --- | --- |
| Age | 1.2 |
| Gender | 1.5 |
| weight | 1.2 |
| Smoking | 1.6 |
| Drinking | 1.5 |
| Hypertension | 1.2 |
| Diabetes | 1.1 |
| Pulmonary diseases | 1.1 |
| Neoadjuvant chemotherapy | 1.3 |
| Hb | 1.6 |
| ALB | 1.2 |
| ASA Status | 1.1 |
| Tumor location | 1.1 |
| Type of anesthesia | 4.7 |
| Continuous anesthesia | 1.1 |
| Vasoactive drug use | 1.1 |
| Surgery method | 3.6 |
| Operation time | 1.7 |
| Perioperative fluid volume | 1.4 |
| Estimated blood loss | 1.8 |
| Patient controlled analgesia | 4.8 |

Dependent variable Y

Y= postoperative pneumonia
Supplementary table 2. Univariate analysis of covariates vs postoperative pneumonia

| Covariates | N | term | beta | Se. | exp(beta) | 95%CI Low | 95%CI Upp | P.value |
| --- | --- | --- | --- | --- | --- | --- | --- | --- |
| Age | 647 | age | 0.0180 | 0.0104 | 1.0182 | 0.9977 | 1.0390 | 0.0824 |
| Gender | 647 | male | 0.6920 | 0.2207 | 1.9977 | 1.2963 | 3.0786 | 0.0017 |
| Weight | 647 | weight | 0.0073 | 0.0091 | 1.0073 | 0.9895 | 1.0255 | 0.4220 |
| Smoking | 647 | smoking | 0.8381 | 0.1744 | 2.3120 | 1.6425 | 3.2543 | <0.0001 |
| Hypertension | 647 | hypertension | 0.0304 | 0.2290 | 1.0309 | 0.6580 | 1.6150 | 0.8943 |
| Drinking | 647 | drinking | 0.4977 | 0.1801 | 1.6449 | 1.1558 | 2.3410 | 0.0057 |
| Diabetes | 647 | diabetes | -0.5000 | 0.3552 | 0.6066 | 0.3024 | 1.2168 | 0.1593 |
| Pulmonary diseases | 647 | pulmonary diseases | 0.4514 | 0.1927 | 1.5705 | 1.0764 | 2.2913 | 0.0192 |
| Neoadjuvant chemotherapy | 647 | Neoadjuvant chemotherapy | 0.2592 | 0.1982 | 1.2959 | 0.8787 | 1.9111 | 0.1910 |
| ASA | 647 | ASA II | -0.5827 | 0.4584 | 0.5584 | 0.2274 | 1.3712 | 0.2036 |
|  |  | ASA III | -0.0650 | 0.5280 | 0.9370 | 0.3329 | 2.6378 | 0.9020 |
| Tumor location | 647 | Middle | -0.4972 | 0.3197 | 0.6082 | 0.3250 | 1.1380 | 0.1198 |
|  |  | Lower | -0.4061 | 0.3610 | 0.6662 | 0.3284 | 1.3517 | 0.2605 |
| Hb | 647 | Hb | 0.0004 | 0.0056 | 1.0004 | 0.9895 | 1.0113 | 0.9491 |
| ALB | 647 | ALB | -0.0213 | 0.0203 | 0.9789 | 0.9408 | 1.0186 | 0.2932 |
| Type of anesthesia | 647 | GA | -0.0906 | 0.1867 | 0.9134 | 0.6335 | 1.3170 | 0.6275 |
| Continuous anesthesia | 647 | TIVA | 0.6505 | 0.2736 | 1.9164 | 1.1209 | 3.2766 | 0.0175 |
| Vasoactive drug use | 647 | Vasoactive drug use | 0.3762 | 0.1694 | 1.4567 | 1.0452 | 2.0303 | 0.0263 |
| Surgery method | 647 | open esophagectomy | 0.2638 | 0.1690 | 1.3018 | 0.9347 | 1.8131 | 0.1186 |
| Estimated blood loss | 647 | Blood loss>200ml | 0.0705 | 0.1738 | 1.0730 | 0.7633 | 1.5084 | 0.6851 |
| Perioperative fluid volume | 647 | perioperative fluid volume | -0.0001 | 0.0001 | 0.9999 | 0.9996 | 1.0002 | 0.4951 |
| Operation time | 647 | operation time | 0.0007 | 0.0015 | 1.0007 | 0.9979 | 1.0036 | 0.6086 |
| PCA | 647 | PCIA | -0.1585 | 0.1869 | 0.8534 | 0.5917 | 1.2309 | 0.3962 |

Supplementary table 3. Add covariates to basic model or remove it from full model, check coeff of X
X= Type of endotracheal tube

|  |  | Basic model | Full model |  |
| --- | --- | --- | --- | --- |
| Covariate | +/- term | SLET | SLET | Selected |
|  | Initial X coeff. | -0.4897 | -0.8746 |  |
| Age | age | -0.5515 * | -0.8221 | Yes |
| Gender | gender | -0.4580 | -0.8582 |  |
| Weight | weight | -0.5055 | -0.8496 |  |
| Smoking | smoking | -0.4208 * | -0.9716 * | Yes |
| Drinking | drinking | -0.4749 | -0.8708 |  |
| Hypertension | hypertension | -0.4966 | -0.8726 |  |
| Pulmonary diseases | pulmonary diseases | -0.4679 | -0.8879 |  |
| Neoadjuvant chemotherapy | neoadjuvant chemotherapy | -0.5669 * | -0.8357 | Yes |
| Hb | Hb | -0.4908 | -0.8736 |  |
| ALB | ALB | -0.5155 | -0.8749 |  |
| ASA | ASA | -0.5031 | -0.8764 |  |
| Tumor location | Tumor location | -0.4863 | -0.8692 |  |
| Type of anesthesia | Type of anesthesia | -0.5546 * | -0.8670 | Yes |
| Continuous anesthesia | Continuous anesthesia | -0.5534 * | -0.8800 | Yes |
| Vasoactive drug use | Vasoactive drug use | -0.6458 * | -0.7606 * | Yes |
| Surgery method | Surgery method | -0.7510 * | -0.8006 | Yes |
| Operation time | Operation time | -0.5643 * | -0.9156 | Yes |
| Estimated blood loss | Estimated blood loss | -0.5681 * | -0.8733 | Yes |
| Perioperative fluid volume | Perioperative fluid volume | -0.4838 | -0.8785 |  |
| Patient controlled analgesia | Patient controlled analgesia | -0.5243 | -0.8963 |  |

* >10% change compare to initial X coeff.

Supplementary table 4. Selected covariates

| Y | X | Selected based on criteria 1 | Selected based on criteria 2 |
| --- | --- | --- | --- |
| Postoperative pneumonia | Type of endotracheal tube | Age,  Smoking,  Neoadjuvant chemotherapy, Type of anesthesia, Continuous anesthesia, Vasoactive drug use, Surgery method,  Operation time,  Estimated blood loss. | Age,  Gender,  Smoking,  Drinking,  Pulmonary diseases,  Neoadjuvant chemotherapy,  Type of anesthesia,  Continuous anesthesia, Vasoactive drug use,  Surgery method,  Operation time,  Estimated blood loss. |

Notes:
1. Criteria 1：add the covariate to basic model or remove it from full model, change X coeff. >10%
2. Criteria 2：criteria 1 or the covariate P<0.1 in univariate model vs Y
