# Supplementary material for: Association of DLT versus SLT with postoperative pneumonia during esophagectomy in China: a retrospective comparison study
Source: BMC Anesthesiol. 2023 Sep 5;23:301. doi: 10.1186/s12871-023-02252-4 (PMC10478392; doi:10.1186/s12871-023-02252-4)

**Supplementary table 5. A timetable on the use of different endotracheal tube**

| **Year** | **Total** | **DLT** | **SLT** | **Pneumonia** | **Pneumonia(%)** |
| --- | --- | --- | --- | --- | --- |
| 2010 | 79 | 77 | 2 | 7 | 8.86% |
| 2011 | 59 | 59 | 0 | 8 | 13.56% |
| 2012 | 74 | 74 | 0 | 35 | 47.3% |
| 2013 | 54 | 54 | 0 | 20 | 37.04% |
| 2014 | 62 | 56 | 6 | 33 | 53.23% |
| 2015 | 65 | 37 | 28 | 32 | 49.23% |
| 2016 | 51 | 15 | 36 | 26 | 50.98% |
| 2017 | 56 | 11 | 45 | 18 | 32.14% |
| 2018 | 55 | 6 | 49 | 12 | 21.82% |
| 2019 | 61 | 4 | 57 | 18 | 29.51% |
| 2020 | 31 | 0 | 31 | 3 | 9.68% |
| Total | 647 | 393 | 254 | 212 | 32.77% |

**Supplementary table 6. The detail of timetable on the use of different endotracheal tube and the occurrence of pneumonia**

| **Year** | **DLT** | | **SLT** | |
| --- | --- | --- | --- | --- |
|  | **Total** | **Pneumonia,n(%)** | **Total** | **Pneumonia,n(%)** |
| 2010 | 77 | 7 (9.09%） | 2 | 0(0%) |
| 2011 | 59 | 8 (13.56%） | 0 | 0 |
| 2012 | 74 | 35 (47.3%) | 0 | 0 |
| 2013 | 54 | 20 (37.04%) | 0 | 0 |
| 2014 | 56 | 30 (53.57%) | 6 | 3 (50%) |
| 2015 | 37 | 24 (64.87%) | 28 | 8 (28.57%) |
| 2016 | 15 | 11 (73.33%) | 36 | 15 (41.67%) |
| 2017 | 11 | 6 (54.55%) | 45 | 12 (26.67%) |
| 2018 | 6 | 2 (33.33%) | 49 | 10 (20.41%) |
| 2019 | 4 | 2 (50%) | 57 | 16 (28.07%) |
| 2020 | 0 | 0 | 31 | 3 (9.68%) |

**Supplementary figure 1. Pneumonia in two groups from 2010 to 2020**


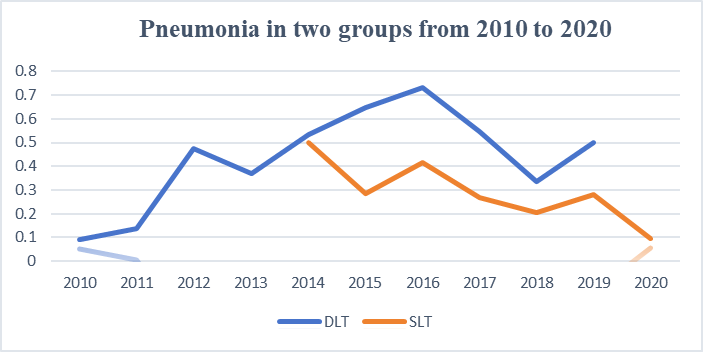

Supplement: Supplementary file 2 — Additional file 2: Supplementary Table 5. A timetable on the use of different endotracheal tube. Supplementary Table 6. The detail of timetable on the use of different endotracheal tube and the occurrence of pneumonia. Supplementary Figure 1. Pneumonia in two groups from 2010 to 2020. [file 12871_2023_2252_MOESM2_ESM.docx]
